# ^18^ F-FDG PET/MR imaging in patients with suspected liver lesions: Value of liver-specific contrast agent Gadobenate dimeglumine

**DOI:** 10.1371/journal.pone.0180349

**Published:** 2017-07-06

**Authors:** Julian Kirchner, Lino M. Sawicki, Cornelius Deuschl, Johannes Grüneisen, Karsten Beiderwellen, Thomas C. Lauenstein, Ken Herrmann, Michael Forsting, Philipp Heusch, Lale Umutlu

**Affiliations:** 1 Department of Diagnostic and Interventional Radiology, University Dusseldorf, Medical Faculty, Dusseldorf, Germany; 2 Institute of Diagnostic and Interventional Radiology and Neuroradiology, University Hospital Duisburg-Essen, Germany; 3 Department of Nuclear Medicine, University Hospital Essen, University of Duisburg-Essen, Essen, Germany; West German Cancer Center, GERMANY

## Abstract

**Objectives:**

To evaluate the added value of the application of the liver-specific contrast phase of Gadobenate dimeglumine (Gd-BOPTA) for detection and characterization of liver lesions in ^18^F-FDG PET/MRI.

**Methods:**

41 patients with histologically confirmed solid tumors and known / suspected liver metastases or not classifiable lesions in ^18^F-FDG PET/CT were included in this study. All patients underwent a subsequent Gd-BOPTA enhanced ^18^F-FDG PET/MRI examination. MRI without liver-specific contrast phase (MRI_1_), MRI with liver-specific contrast phase (MRI_2_), ^18^F-FDG PET/MRI without liver-specific contrast phase (PET/MRI_1_) and with liver-specific contrast phase (PET/MRI_2_) were separately evaluated for suspect lesions regarding lesion dignity, characterization, conspicuity and confidence.

**Results:**

PET/MRI datasets enabled correct identification of 18/18 patients with malignant lesions; MRI datasets correctly identified 17/18 patients. On a lesion-based analysis PET/MRI_2_ provided highest accuracy for differentiation of lesions into malignant and benign lesions of 98% and 100%. Respective values were 95% and 100% for PET/MRI_1_, 93% and 96% for MRI_2_ and 91% and 93% for MRI_1_. Statistically significant higher diagnostic confidence was found for PET/MRI_2_ and MRI_2_ datasets compared to PET/MRI_1_ and MRI_1,_ respectively (p < 0.001).

**Conclusion:**

The application of the liver-specific contrast phase in ^18^F-FDG PET/MRI further increases the diagnostic accuracy and diagnostic confidence for correct assessment of benign and malignant liver lesions.

## Introduction

As the second most frequent organ manifestation of distant metastases [1], liver metastases are considered among the most common malignant liver lesions and occur approximately up to 18–40 times more frequent than primary liver malignancies [2]. Malignancies accounting for liver metastases are primarily lung cancer, breast cancer, colorectal cancer, and pancreatic carcinoma [3]. While the incidence of liver metastases significantly outnumbers the occurrence of hepatocellular carcinoma (HCC), HCC accounts for up to 80% of primary liver cancers [4] and is regarded as the sixth most common cancer worldwide and the third most common cause of cancer-related death [5]. Correct determination of the hepatic tumor-spread is crucial for optimized therapy stratification as potentially curative options are commonly restricted to resection. Furthermore, apart from malignant liver pathologies, benign lesions also demand correct identification as some liver lesions e.g. adenomas are known to be associated with potential complications such as hemorrhage, rupture or malignization [6–8]. Hence, high-quality liver imaging is crucial for best patient care and therapy management. While computed tomography (CT) and transabdominal ultrasound still represent the primary and most commonly applied imaging modalities, more advanced imaging techniques such as magnetic resonance imaging (MRI) have been shown superior for correct depiction and characterization of liver lesions when compared to ultrasound, CT and positron emission tomography / CT using ^18^F-fluoredeoxyglucose [9–11]. Particularly the utilization of liver-specific contrast agents has been shown to provide important additional diagnostic information for assessment of small lesions < 15mm, hepatocellular carcinoma as well as for correct discrimination of liver adenomas from focal nodular hyperplasias (FNH) [12–15]. Hence, in accordance with the ESGAR consensus statement on liver MR imaging the application of liver-specific contrast agents is recommended in patients considered for liver resection [16]. Apart from its excellent diagnostic capacity, MR imaging is restricted to morphological and functional information of lesions, lacking the metabolic component of PET imaging. With the successful introduction of integrated PET/MRI systems into clinical imaging [17], numerous studies have demonstrated the high diagnostic quality and potential superiority of PET/MRI in dedicated applications [18–21]. Reporting initial results, Beiderwellen et al demonstrated higher lesion conspicuity and diagnostic confidence in liver lesions in PET/MRI compared to PET/CT in a whole body staging approach [22]. Recent publications also investigated the added value of the utilization of liver-specific contrast agents in PET/MRI for detection of malignant liver lesions [23]. Thus, following these first results, the purpose of this study was to evaluate the added value of the liver-specific contrast phase of Gadobenate dimeglumine (MultiHance, Bracco, Milano, Italy) enhanced ^18^F-FDG PET/MRI for detection and characterization of malignant and benign liver lesions.

## Material & methods

### Patients

The study was conducted in conformance with the Declaration of Helsinki and approved by the Ethics Commission of the Medical Faculty of the University Duisburg-Essen (study number 11-4822-BO). All patients underwent a clinically indicated whole-body ^18^F-FDG-PET/CT (PET/CT) and subsequently an additional ^18^F-FDG PET/MRI (PET/MRI) of the liver with application of Gadobenate dimeglumine (Gd-BOPTA) after informed written consent was obtained.

41 patients (mean age 55.9 ± 14.5 years; range 27–79) met the inclusion criteria of histologically confirmed solid tumors and known or suspected liver metastases or non-classifiable lesions in PET/CT (Table 1). Exclusion criteria were patient age <18 years and contraindication to MRI such as pacemaker or chronic renal failure. Primary tumors included primary liver tumors as well as extrahepatic tumors (Table 2).

**Table 1:** Demographic characteristics.

sex	number	mean age (years)
female	21	56.4 ± 16
male	20	54.3 ± 12.8

**Table 2:** List of primary tumors.

	*n*	*%*
Colorectal Carcinoma	19	46
Hepatocelluar Carcinoma	4	10
Melanoma	4	10
Cholangiocellular Carcinoma	3	7
Breast Cancer	2	5
Other	9	22
Total	41	100

### PET/MRI

^18^F-FDG PET/MRI examinations were performed on an integrated 3 Tesla PET/MRI scanner (Biograph mMR, Siemens Healthcare GmbH, Erlangen, Germany) subsequently after clinically indicated ^18^F-FDG PET/CT examinations and obtained with an average delay of 153 ± 35 min (range: 96 min—138 min) after intravenous injection of body-weight adapted mean activity of 244 MBq ± 53 MBq (range: 106 Mbq—382 Mbq) ^18^F-FDG. No additional tracer was injected for the subsequent ^18^F-FDG PET/MRI examinations. The liver-specific contrast phase was acquired on average 74 ± 25 (range 58–122 min) after contrast media application. Scan volumes covered the whole liver.

**Fig 1:**
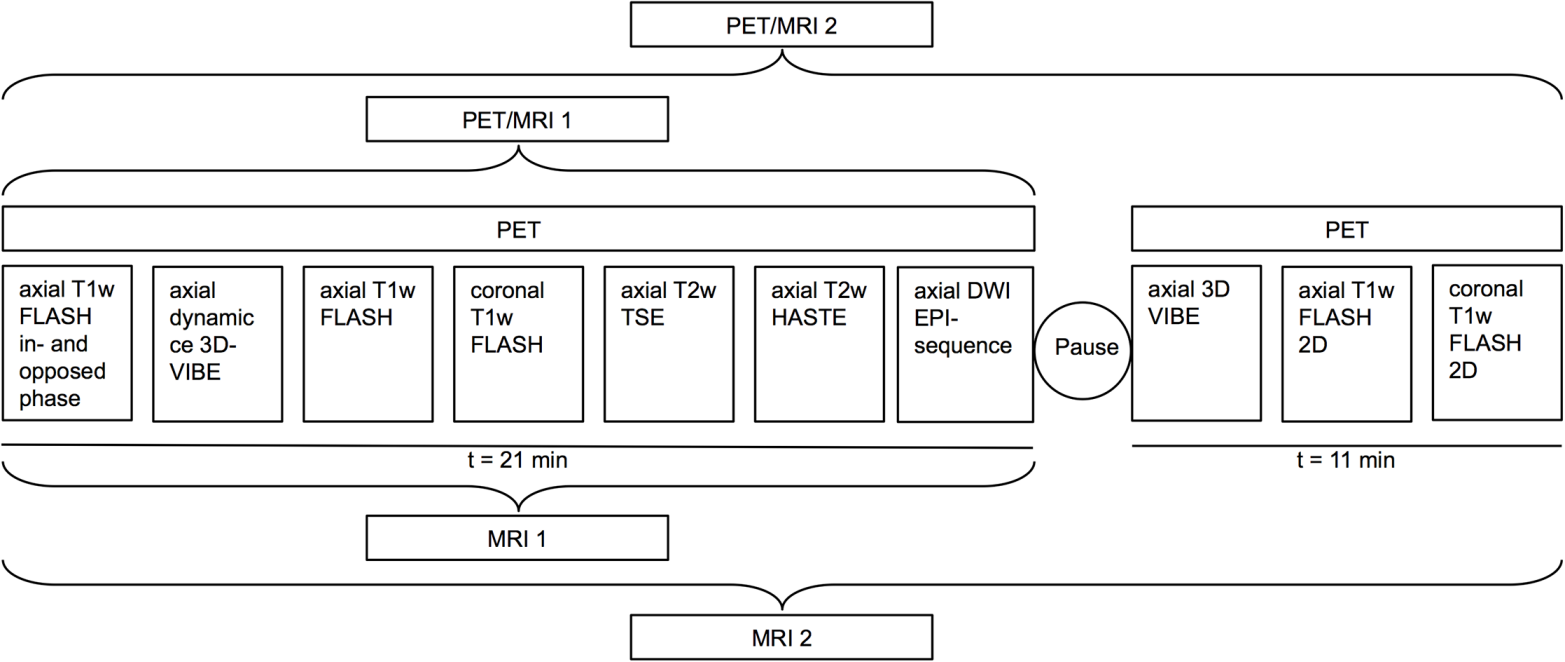
Overview of the imaging protocol and timing of each image acquisition. T1w VIBE = T1-weighted Volume-Interpolated Breath-hold Examination; T2w HASTE = T2-weighted Half Fourier Acquisition Single Shot Turbo Spin Echo; T1w FLASH = T1-weighted Fast Low-angle Shot; T2w TSE = T2-weighted Turbo-spin Echo; DW EPI = Diffusion-weighted Echo-Planar imaging.

PET data acquisition was performed for early phase datasets as well as for liver-specific contrast phase datasets in 1 bed position with 10 minutes. PET images were reconstructed using the iterative ordered-subset expectation maximization (OSEM) algorithm, 3 iterations and 21 subsets, a Gaussian filter with 4mm full width at half maximum (FWHM) and a 344 × 344 image matrix. For MR-based signal intensity correction a two-point (fat, water) coronal 3D-Dixon-VIBE (volumetric interpolated breath-hold examination) sequence was performed to generate a four-compartment model (background air, lungs, fat, muscle) [24]. The pre-contrast MRI sequences were obtained simultaneously using a 24-channel spine-array radiofrequency coil as well as 16-channel flex body-coils, depending on patient size. After completion of PET-acquisition, additional Gd-BOPTA enhanced sequences were acquired. The following imaging protocol was applied (Fig 1):

1 A coronal T1-w 3D-Dixon-VIBE sequence prior contrast administration for attenuation correction only.
2 Am axial T1-w FLASH (fast low-angle shot) in- and opposed phase in breath-hold technique with a slice thickness of 7 mm (Echo Time [TE] 2 & 3.4 ms; Repetition Time [TR] 111 ms; Field of View [FOV] 400 mm; phase FOV 75%; acquisition matrix 256 × 192; in plane resolution 0.8 × 0.8 mm)
3 An axial dynamic contrast-enhanced 3-dimensional Volumetric Interpolated Breath-hold Examination (VIBE) with a slice thickness of 3.5 mm (TE, 1.5 ms; TR, 4 ms; Flip angle 9◦; FOV 400 mm; phase FOV 75%; acquisition matrix 512 × 384, in plane resolution 0.8 x 0.8 mm) before and 20s, 50s and 80s after intravenous administration of Multihance (0,05 mmol/kg bw)
4 An axial T1-w FLASH 2D fat-saturated in breath-hold technique with a slice thickness of 7mm (TE, 3.62 ms; TR 185 ms; FOV 400 mm; phase FOV 75%; acquisition matrix 320 × 240, in plane resolution 1.3 x 1.3 mm)
5 A coronal T1-w FLASH 2D fat-saturated in breath-hold technique with a slice thickness of 6 mm (TE, 2.49 ms; TR 125 ms; FOV 360 mm; phase FOV 100%; acquisition matrix 256 × 256, in plane resolution 1.4 x 1.4 mm)
6 An axial T2-w TSE (Turbo-spin Echo) fat-saturated in breath-hold technique with a slice thickness of 7 mm (TE 97 ms; TR 2840 ms; FOV 400 mm; phase FOV 750%; acquisition matrix 256 × 192, in plane resolution 1.6 x 1.6 mm)
7 An axial T2-w HASTE (half Fourier acquisition single shot turbo spin echo) in breath-hold technique with a slice thickness of 7 mm (TE 100 ms; TR 1000 ms; Turbo factor (TF) 194; FOV 400 mm; phase FOV 75%; acquisition matrix 320 × 240 mm; in plane resolution 1.3 x 1.3 mm)
8 An axial diffusion-weighted echo-planar imaging (EPI) sequence in free breathing with a slice thickness of 5.0 mm (TR 8000 ms; TE 81 ms; b-values: 0, 500 and 1000 s/mm2, matrix size 192 x 156; FOV 420 mm, phase FOV, 81.3%; GRAPPA, acceleration factor 2; in plane resolution 2.2 x 2.2 mm)

Pause and subsequently acquired liver-specific phase.

9 A coronal T1-w 3D-Dixon-VIBE sequence for attenuation correction only.
10 An axial 3D VIBE with a slice thickness of 3.5 mm (TE 1.5 ms; TR 4 ms; Flip angle 9◦; FOV 400 mm; phase FOV 75%; acquisition matrix 512 × 384, in plane resolution 0.8 x 0.8 mm).
11 An axial T1w FLASH 2D fat-saturated in breath-hold technique with a slice thickness of 7 mm (TE 3.62 ms; TR 185 ms; FOV 400 mm; phase FOV 75%; acquisition matrix 320 × 240, in plane resolution 1.3 x 1.3 mm)
12 A coronal T1w FLASH 2D fat-saturated in breath-hold technique with a slice thickness of 6 mm (TE 2.49 ms; TR 125 ms; FOV 360 mm; phase FOV 100%; acquisition matrix 256 × 256, in plane resolution 1.4 x 1.4 mm)

### Image analysis

The following imaging datasets of the ^18^F-FDG PET/MR examination were analysed in consensus and in random order by two experienced radiologists in hybrid and MR imaging interpretation on a dedicated OsiriX Workstation (Pixmeo SARL, Bernex, Switzerland): MRI without liver-specific contrast phase (MRI_1_), MRI with liver-specific contrast phase (MRI_2_), ^18^F-FDG PET/MRI without liver-specific contrast phase (PET/MRI_1_) and with liver-specific contrast phase (PET/MRI_2_). All datasets were evaluated in two reading sessions in random order and both readers were blinded to patient identity and results of prior or follow-up imaging. In a first reading session radiologists evaluated MRI_1_ and subsequently MRI_2_. In a second reading session with a minimum of four weeks apart to avoid recognition bias, PET/MRI_1_ and subsequently PET/MRI_2_ were evaluated. Interpretation was performed patient- and lesion-based. In conformity to previous studies [22] every liver lesion in each patient was rated with regard to lesion dignity (1, benign; 2, malignant), lesion conspicuity (5-point ordinal scale: 1, not visible; 2, very low contrast; 3, low contrast; 4, intermediate contrast; 5, high contrast) and diagnostic confidence (5-point ordinal scale: 1, very low confidence; 2, low confidence; 3, indeterminate confidence; 4, high confidence; 5, very high confidence).

Gadobenate dimeglumine differs from extracellular gadolinium agents as a fraction of 3–5% of the injected dose is taken up into functioning hepatocytes, entailing long-lasting enhancement of the normal liver parenchyma that results in significantly increased sensitivity and characterization for liver lesions in T1-weighted images between 40 and 120 minutes after intravenous administration [25,26]. Hence, Gadobenate dimeglumine and other so-called liver-specific contrast agents have been demonstrated to enable an improved detection and differentiation of small metastases and HCCs as well as an improved differentiation of benign liver lesions, such as liver adenomas from FNH [27]. According to the ESGAR consensus statement on liver, MRI liver-specific contrast agents are recommended to be applied in pre-surgical work up of hepatic lesions for lesion detection and characterization, particularly when the differential diagnosis is primarily between solid benign lesion (e.g. FNH) versus metastasis and for clear delineation of primary liver tumors [16]. As metastatic involvement of the liver is known to lead to major changes in therapy management, correct identification of the extent of hepatic tumor spread is of utmost importance in a pre-therapeutic setting as well as in a post-surgical setting since incomplete resection does not prolong survival [28] (Fig 1E–1H). Furthermore, in contrary to most benign liver lesions, that do not require resection or follow-up, liver adenomas demand correct characterization, as they are known to bear the risk of spontaneous rupture or degeneration to hepatocellular carcinoma [29].

**Fig 2:**
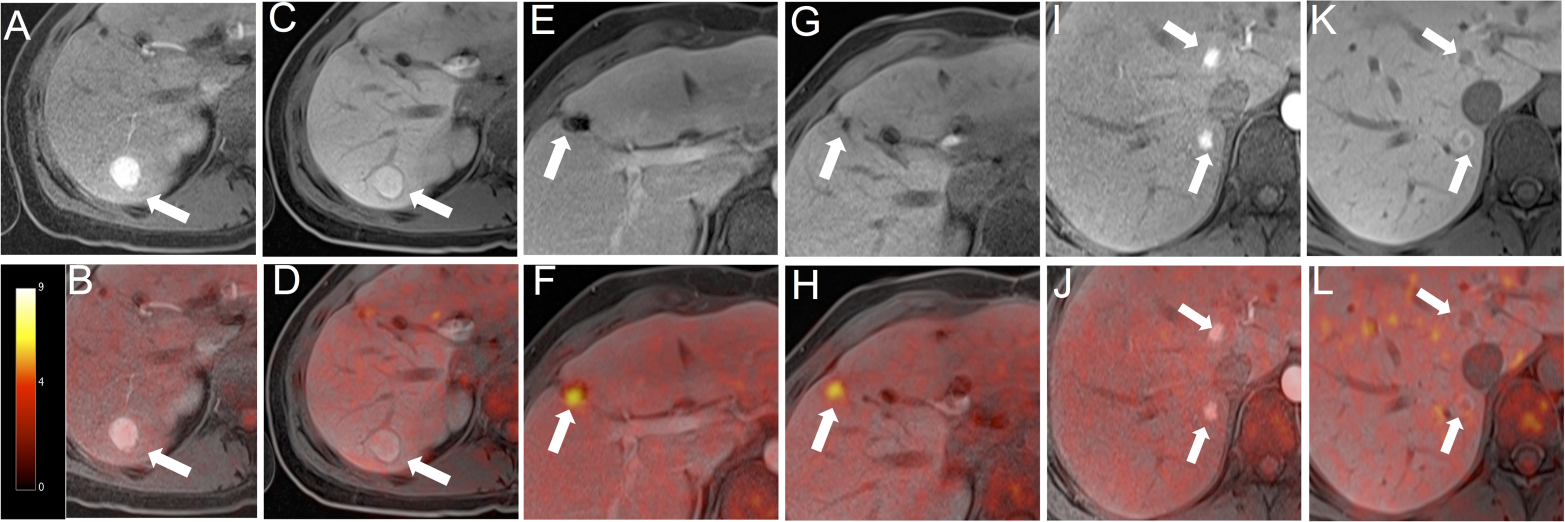
A 25-year-old female patient with a history of colorectal cancer presented multiple liver lesions after surgery. The FNH in the right liver shows an arterial contrast-agent enhancement (A) and is still hyperintense in the liver-specific contrast phase (C). No significant ^18^F-FDG-uptake is seen (B, D). A second lesion in the right liver is rated as a colorectal liver metastasis due to incomplete resection. Tumor lesion is neither detectable by MRI without liver-specific contrast phase nor with liver-specific contrast phase (E; G). In fused PET/MR images (F; H) the remaining tumor tissue lesion could clearly be identified. Additional lesions near the liver hilus are adenomas with strong arterial contrast-agent enhancement (I). In the liver-specific contrast phase lesions are hypointense (K). Similar to the FNH, no significant ^18^F-FDG-uptake is seen (J, L).

For the characterization of the focal liver lesions detected in each interpreting session, the readers were asked first to decide whether a lesion was benign or malignant and to then give a diagnosis, in terms of characterization of the lesions. Malignant lesions comprised hepatocellular carcinoma (HCC), cholangiocellular carcinoma (CCC) and metastasis. Benign lesions comprised cyst, haemangioma, focal nodular hyperplasia, adenoma and regenerating nodule (Fig 2). The readers were given information about the primary tumor localization, as liver metastases originating from different tumors may show different contrast enhancement patterns. Lesion characterization was performed based on all available T1 and T2 weighted sequences as well as DWI. A potential diffusion restriction with corresponding signal drop in the ADC map was considered indicative for malignancy.

For lesion characterization on PET, visually increased focal ^18^F-FDG-uptake in comparison to surrounding tissue was considered indicative of malignancy (PET-positive lesions). In accordance with previous publications lesions were rated as metastases when at least two of the three following criteria in MRI_1_ and PET/MRI_1_ or rather three of the four following criteria in in MRI_2_ and PET/MRI_2_ were found: (1) Hyperintense lesion in T2w images with ill-defined borders, (2) diffusion restriction on DWI, (3) contrast behaviour not inkeeping with cysts, hemangioma, FNH or adenoma and (4) hypointense lesion in liver-specific phase [30,31].

In all lesions demonstrating focal ^18^F-FDG uptake the maximum standardized uptake value (SUVmax) was measured by placing a manually drawn polygonal volume of interest (VOI) over each lesion on attenuation-corrected PET images. Additionally, the maximum discernible diameters of all suspicious lesions were determined in the liver-specific contrast phase. In accordance with previous publications reporting the superiority of PET uptake over morphology [32] as well as the high sensitivity for detection of malignant lesions in MRI when utilizing the liver-specific contrast phase [33], discrepant findings on PET and MR datasets as well as discrepancies between the two readers, a consensus decision among the two readers was made based on all available data.

### Reference standard

Histopathological confirmation of the primary tumors was available in all patients except for the ones suffering from cancer unknown primary (CUP). In accordance with current treatment guidelines and ethical considerations, histopathological correlation for each depicted suspicious liver lesion was not clinically indicated and hence not available. A consensus characterization for each lesion based on available prior examinations, histopathological data, PET/CT, PET/MRI as well as imaging and clinical follow-up served as the standard of reference (mean interval of 295 ± 343 days).

### Statistical analysis

Statistical analysis was performed using IBM SPSS version 22 (IBM Inc, Armonk, NY, USA).

Data analysis was performed patient-based as well as lesion based. The scores of resulting datasets were analysed first on a descriptive basis. Due to the ordinal scale scores the median scores were subsequently compared with the Friedman test. As post hoc test Wilcoxon signed-rank test was chosen and Bonferroni adjustment was applied.

## Results

### Patient based analysis

Based on the reference standard liver lesions were present in 36 / 41 patients (88%). Malignant liver lesions were present in 18 patients (44%), benign lesions in 27 patients (66%). Based on MRI alone (MRI_1_ and MRI_2_) 17 patients (94%) with malignant lesions could be correctly identified with one false-positive rating (4%). Both PET/MRI datasets (PET/MRI_1_ and PET/MRI_2_) enabled a correct identification of all 18 patients (100%) with malignant liver lesions.

### Lesion-based analysis

**Fig 3:**
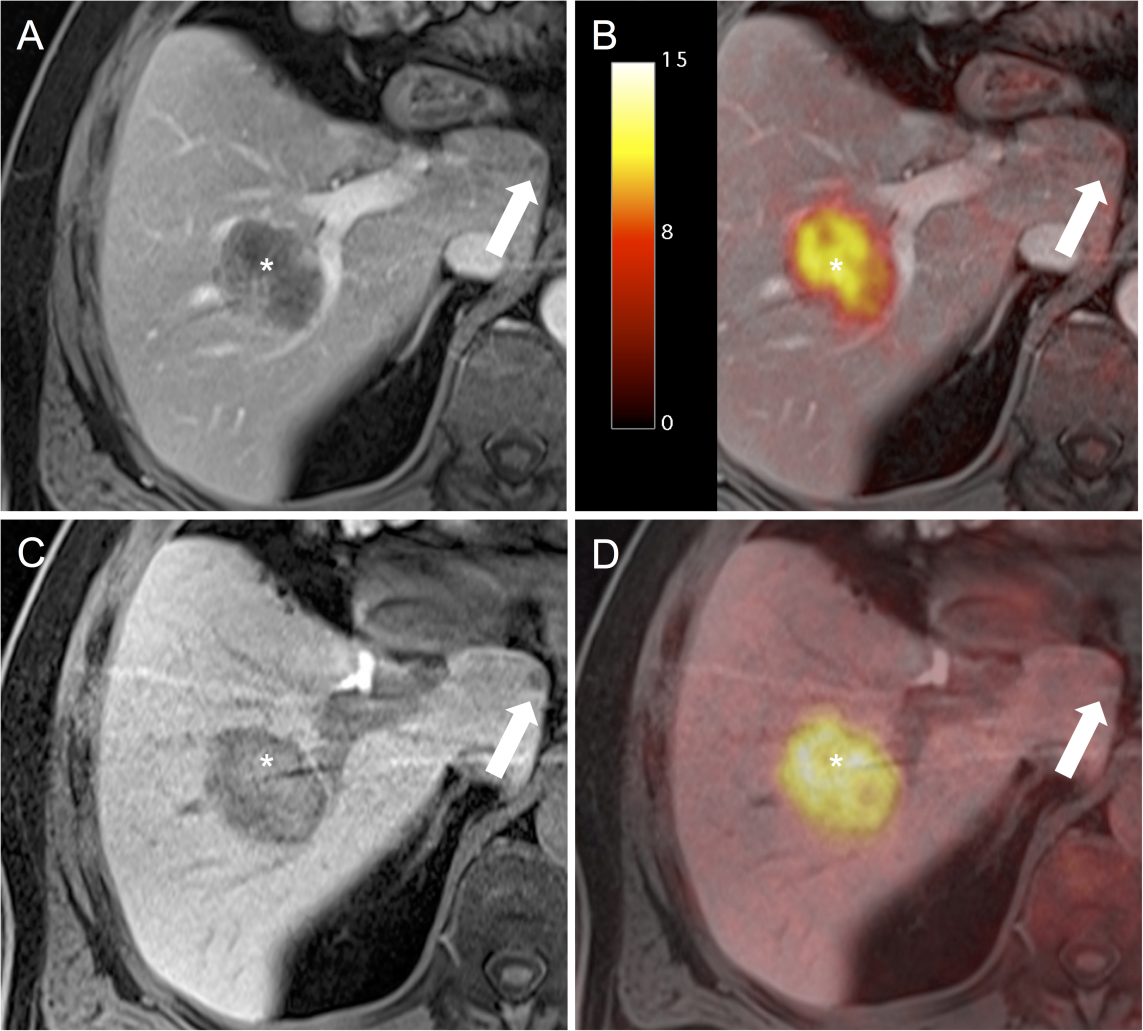
A 61-year-old male patient with liver metastases from a Colorectal Carcinoma. The large metastasis with intense ^18^F-FDG-uptake in the right central liver lobe is clearly visible in all datasets (*). The metastasis in the Lobus caudatus does not show increased ^18^F-FDG-uptake (A, D) and is hardly detectable in the arterial-phase (B) but is clearly detectable as hypointense lesion in the liver-specific phase (C).

Based on the reference standard a total of 137 lesions were detected. These comprised 80 (58%) benign and 57 (42%) malignant lesions (Table 3). All lesions could be detected in the readings including the liver-specific contrast phase (MRI_2_ and PET/MRI_2_), while one lesion was missed in the readings without the liver-specific contrast phase (MRI_1_ and PET/MRI_1_). The missed lesion in the datasets without the liver-specific contrast phase was due to a 6 mm, subcapsular lesion without FDG-uptake, that was only clearly discernible based on its hypointense appearance in the liver-specific phase (Fig 3). Highest accuracy for differentiation between malignant and benign lesions was achieved based on the PET/MRI_2_ datasets, entailing a correct classification of 98% of the malignant and 100% of the benign lesions. The respective values for PET/MRI_1_ were 95% and 100%, for MRI_2_ 93% and 96% and 91% and 93% for MRI_1_ (Table 4). The missed lesion in PET/MRI_2_ was due to a 7 mm lesion without significant tracer uptake or clear diffusion restriction, which was falsely rated as a hemangioma and emerged as a metastasis in follow-up imaging.

**Table 3:** Lesion character in benign liver lesions in accordance to reference standard.

	*n*	*%*
Liver cyst	42	52.5
Hemangioma	11	13.75
Focal nodular hyperplasia	9	11.25
Scar tissue	6	7.5
Avital metastases	5	6.25
Adenoma	4	5
Regenerative nodule	2	2.5
Inflammatory	1	1.25
Total	80	100

**Table 4:** PET/MRI with liver specific-contrast phase (PET/MRI_2_) offers highest accuracy for lesion classification in malignant and benign compared to PET/MRI without liver-specific phase (PET/MRI_1_) and MRI with (MRI_1_) and without liver-specific phase (MRI_2_).

Lesions	MRI_1_ (%)	MRI_2_ (%)	PET/MRI_1_ (%)	PET/MRI_2_ (%)
Malignant *n* = 57	52 (91)	53 (93)	54 (95)	56 (98)
Benign *n* = 80	77 (96)	77 (96)	80 (100)	80 (100)
Total *n* = 137	129 (94)	130 (95)	134 (98)	136 (99)

Total numbers and the percentage (in parentheses) of correct classification in malignant and benign lesion for every modality are given.

Based on both readings including the liver specific phase (PET/MRI_2_ and MRI_2_) correct classification of all (100%) benign lesions was possible, while the exclusion of the liver-specific contrast phase (PET/MRI_1_ and MRI_1_) enabled a correct classification of 96% of the benign lesions (Table 5). Misclassifications in datasets without liver-specific phase were due to mischaracterization of FNH and adenoma.

**Table 5:** Correct classification of benign lesion in the different modalities in percentage.

	MRI_1_	MRI_2_	PET/MRI_1_	PET/MRI_2_
in %	96	100	96	100

### Diagnostic confidence & lesion conspicuity

**Fig 4:**
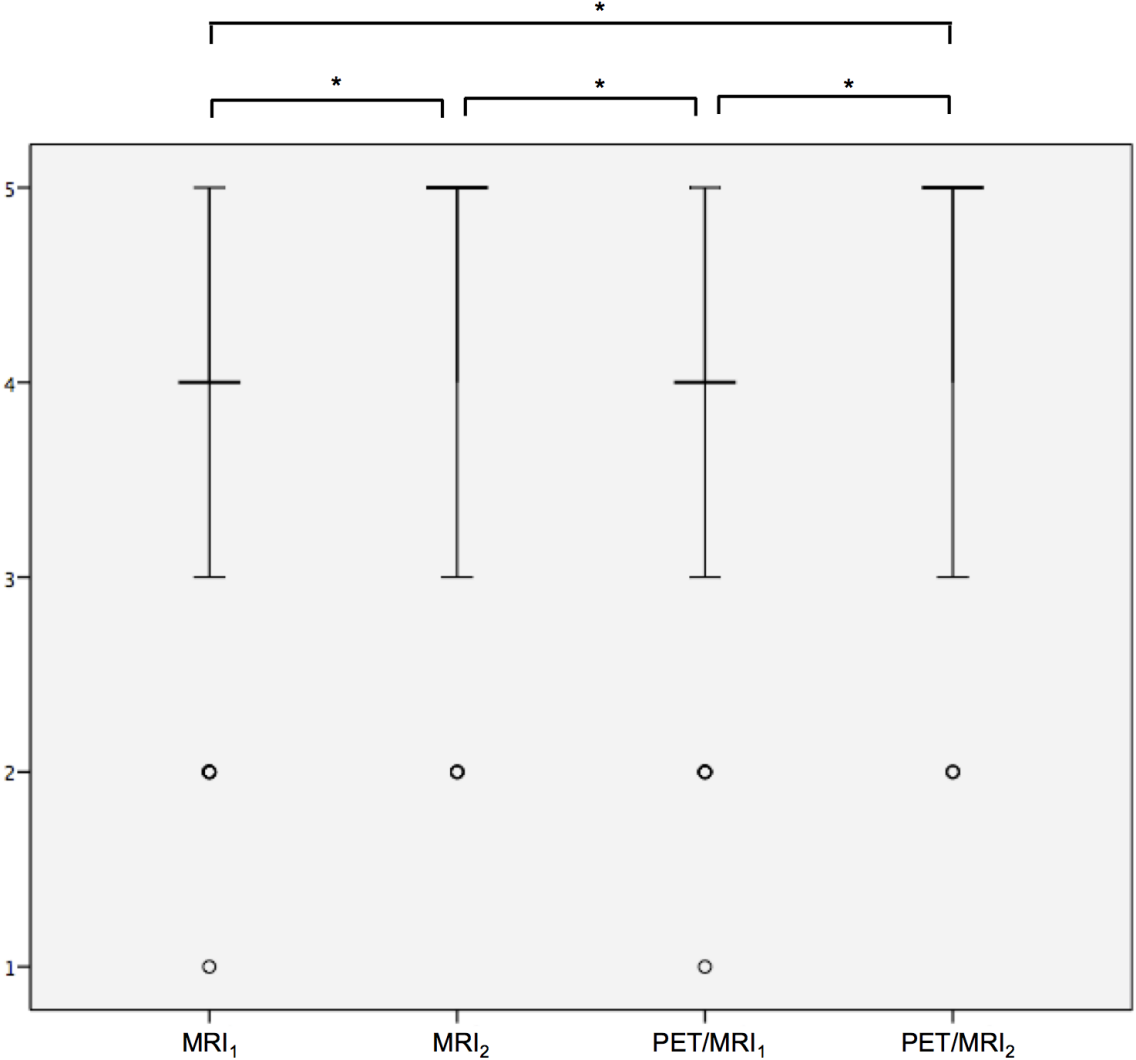
Diagnostic confidence is significantly higher in PET/MRI_2_ and MRI_2_ datasets compared to PET/MRI_1_ and MRI. Ratings for lesion conspicuity are entered on the y-axis. The vertical bar represents the upper and lower quartiles; the horizontal bar represents the median. The points represent extreme values. Significant differences are marked by a star.

There was a statistically significant difference in the resulting scores for diagnostic confidence (p < 0.001) (Fig 4). Post hoc analysis with Wilcoxon signed-rank test was conducted with a Bonferroni correction applied, resulting in a significance level set at p < 0.0125. The score for MRI_2_ was significantly higher compared to MRI_1_ (MRI_2_: median 5, range 2–5; MRI_1_: median 4; range 1–5; p < 0.001) as well as to PET/MRI_1_ (PET/MRI_1_: median 4; range 1–5; p < 0.001). The score for PET/MRI_2_ was significantly higher compared to MRI_1_ (PET/MRI_2_: median 5, range 2–5; p < 0.001) and PET/MRI_1_ (p < 0.001). No statistically significant difference was shown between MRI_2_ and PET/MRI_2_ regarding the diagnostic confidence (p = 0.18). No statistically significant difference was detected between the modalities regarding lesion conspicuity (p = 0.88).

## Discussion

Our results demonstrate the feasibility and high diagnostic performance of Gd-BOPTA enhanced ^18^F-FDG PET/MRI and the additional value of the liver-specific contrast phase for depiction and characterization of liver lesions. This publication comprises two main messages we believe to be important: First, the accuracy in differentiating benign from malignant liver lesions in Gd-BOPTA enhanced ^18^F-FDG PET/MRI with an additional liver-specific contrast phase is superior to the accuracy in Gd-BOPTA enhanced ^18^F-FDG PET/MRI without an additional liver-specific contrast phase as well as in Gd-BOPTA enhanced MRI alone with and / or without an additional liver-specific contrast phase. Secondly, the application of a liver-specific contrast phase enables improved differentiation between potentially therapy-requesting liver adenomas and other benign liver lesions.

Hybrid imaging, in terms of PET/MRI, combines the high spatial resolution and soft-tissue contrast of MRI with the metabolic information based on the PET dataset. Recent studies demonstrated the high lesion conspicuity and diagnostic confidence in PET/MRI regarding liver lesions in a whole body approach [22] and its significantly higher diagnostic accuracy in the detection of liver metastases when compared to PET/CT [31].

Initial trials on detection and characterization of liver lesions have focused on the general comparison of PET/MRI and PET/CT [22,31] or MRI and PET/CT [9] as well as the added value of diffusion weighted imaging in liver MRI [34–36] without putting the focus on the value of liver-specific contrast agents. Recent trials have investigated the added value of liver-specific contrast agents for the differentiation of benign and malignant liver lesion in hybrid PET/MR imaging [23,37]. Donati et al reported their results on retrospective PET-MRI fusion in patients undergoing PET/CT and subsequent liver MRI with a liver specific contrast agent (Gd-EOB-DTPA) [37]. Comparable to our results, the authors reported a higher accuracy in detection and correct determination of the lesions dignity in (retrospectively fused) PET/MRI over MRI alone (93% vs. 91%). But Donati et al only divided the lesions in malignant and benign and forego to give the exact diagnosis. Furthermore they omitted the application of diffusion-weighted imaging. A study including this highly-appreciated imaging technique was published by Lee et al [23]. In this study the authors evaluated the diagnostic performance of integrated ^18^F-FDG PET/MRI in the detection of colorectal cancer liver metastases.

Diffusion-weighted imaging has been well-established for liver imaging in the past years, and while it has become a valuable imaging method particularly for the detection of liver metastases, it is still recommended to be used as an adjunct to contrast-enhanced liver MRI in lieu of a stand-alone tool due to significant overlaps between ADC values of benign and malignant lesions [16]. Hence, current guidelines recommend the combined application of DWI and liver-specific enhanced MRI for detection of (small) metastases [16]. Comparable to our results, Lee et al demonstrated the superiority of ^18^F-FDG PET/MRI over MRI (each enhanced with liver-specific contrast agent) for detection of liver metastases, yet failing to merit significant difference. Our study differs in two points from Lee et al. First, in contrary to Lee et al, who only included patients suffering from colorectal carcinoma resulting in a mostly homogenous appearance of suspected malignancies, the patients in our study suffered from a wide range of primary malignancies, resulting in a potentially more diverse and hence more difficult to characterize, delineation of metastases. Secondly, we additionally investigated the diagnostic value of the liver-specific contrast phase not only for detection and differentiation of malignant from benign lesions but also for dedicated characterization of benign lesions. According to our results, the utilization of the liver specific phase enabled the highest diagnostic accuracy for detection and differentiation of malignant from benign lesions in ^18^F-FDG PET/MRI and superior diagnostic accuracy when comparing MRI alone (with liver-specific contrast phase versus without liver-specific contrast phase). Furthermore, the utilization of the liver specific phase also led to an improved differentiation of liver adenomas from FNH, a differentiation with important clinical and therapeutic impact. This goes in line with previous publications underlining the diagnostic benefit of liver specific contrast agents for characterization of benign liver lesions and improvement of the diagnostic confidence. Gazioli et al reported specificity rates for differentiation of FNH, adenoma and liver adenomatosis to be up to 100% based on Gd-BOPTA enhanced MRI with liver-specific contrast phase, while confident differential diagnosis was not possible in 70% on the basic of dynamic phase imaging alone [38].

Our study is not without some limitations. Even though for the primary tumor a histopathological correlation was available, due to ethical reasons a histopathological sampling of each detected lesion was not applicable. To overcome this limitation, a well-established modified reference standard was applied [39,40] comprising all available data based on clinical follow-up, imaging and histopathology.

In conclusion our results demonstrate a further increase in diagnostic accuracy for depiction and characterization of liver lesions based on the application of a liver-specific contrast phase in ^18^F-FDG PET/MR imaging, in addition to standard dynamic contrast-enhanced imaging and DWI. Thus, the application of an enhanced liver imaging protocol in^18^F-FDG PET/MR imaging, including the liver specific phase, may improve therapeutic stratification and patient management.
